# The Spectrum of Parenchymal Changes in Kidneys Affected by Intrarenal Reflux, Diagnosed by Contrast-Enhanced Voiding Urosonography and DMSA Scan

**DOI:** 10.3389/fped.2022.886112

**Published:** 2022-07-11

**Authors:** Ana Simičić Majce, Adela Arapović, Vesna Čapkun, Dubravka Brdar, Marko Brekalo, Ileana Zebić, Ana Barić, Ante Punda, Mirna Saraga-Babić, Katarina Vukojević, Marijan Saraga

**Affiliations:** ^1^Department of Pediatrics, University Hospital Split, Split, Croatia; ^2^Department of Nuclear Medicine, University Hospital Split, Split, Croatia; ^3^School of Medicine, University of Split, Split, Croatia; ^4^Department of Anatomy, Histology and Embryology, School of Medicine, University of Split, Split, Croatia

**Keywords:** vesico ureteral reflux, intrarenal reflux, contrast-enhanced voiding urosonography, radioisotope scanning, children

## Abstract

**Purpose:**

To describe the parenchymal defects in kidneys with intrarenal reflux (IRR) diagnosed using contrast-enhanced voiding urosonography (ceVUS) and ^99m^Tc-DMSA scintigraphy (DMSA scan).

**Materials and Methods:**

A group of 186 uretero-renal units (URUs) was analyzed using ceVUS and DMSA scans: 47 without vesicoureteral reflux (VUR) (group A) and 139 with VURs, comprising 73 VURs without (group B), and 66 with IRR (group C). VURs included non-dilating (grades I–II), mildly non-dilating (grade III), and non-dilating (grades IV–V) grades. The parenchymal changes were analyzed using a DMSA scan.

**Results:**

The median age for VUR diagnosis was 16.5 months in girls, and 8.5 months in boys (*Z* = 3.9; *p* = 0.001). IRR occurred in 51.4% of boys and in 25.9% of girls (χ^2^ = 12.4; *p* < 0.001). The non-dilating VUR occurred in 44% of boys and 24.1% of girls (χ^2^ = 7.7; *p* = 0.005). IRRs characterized upper and lower renal segments (81.8 and 63.6%) and middle segments (33.3%). Both incidence and increase in IRR correlated with the grade of VUR (*p* < 0.001). The incidence of reduced DMSA signal was statistically different among groups A + B and C, but not between groups A and B (χ^2^ = 32.2; *p* < 0.001). No statistically significant relationship existed between the reduced DMSA signal and the grade of VUR in group C. The reduced DMSA signal appeared in 9.9% positions in kidneys from group A, 14% from group B, and 32% from group C. Out of all 118 IRRs, 38.1% had reduced and 61.9% had normal DMSA signal. Among 11 parenchymal scars found in all three groups, 2 belonged to group B, 9 to group C, while group A had no scars.

**Conclusion:**

The parenchymal changes are the most prominent in the group with IRR, but they do not significantly differ among kidneys with different grades of VUR. VURs of higher grades are associated with a higher incidence of IRR and early clinical presentation. Scars can also appear in lower-grade VURs accompanied by IRR. Boys with VUR have earlier clinical presentation than girls, as they have significantly higher grades of VUR with a higher proportion of IRRs. Therefore, we suggest a subdivision of VURs into those with IRR and abundant parenchymal damage, and those without IRR and less parenchymal damage.

## Introduction

Vesicoureteral reflux (VUR) is one of the most common abnormalities of the urinary tract in children, with a prevalence of 1–2% (1). It is defined as a backflow of urine from the urinary bladder to the ureter, pyelo calyceal system, even in the renal parenchyma, and is divided into 5 grades according to voiding cystourethrography (VCUG) and contrast-enhanced voiding urosonography (ceVUS), plus one extra grade detected solely by direct radionuclide cystography (DRNC) (2–4). VUR is the predisposing factor for urinary tract infections (UTI) and renal parenchymal damage in children. The prevalence of parenchymal damage was significantly correlated with reflux grade (5–11). Sometimes the VUR can be intrarenal when the urine reaches the kidney parenchyma and penetrates the tubulointerstitial area through papillary ducts. It can be detected by VCUG (12) and by ceVUS, especially by the second generation of ultrasound contrast agents (UCA) (13, 14).

Intrarenal reflux (IRR) can be the cause of renal parenchymal damage and earlier clinical presentation than in patients with VUR but without IRR (15–20). It is known from the literature that IRRs, detected by VCUG, are highly associated with scar formation in the renal parenchyma on a DMSA scan (17, 18, 20, 21). Some authors detected 1–11% of IRRs among all VURs by using VCUG (17, 20, 22). Such a variable incidence of IRRs was probably dependent on insufficient technique during VCUG. Therefore, Schneider and collaborators believed that the incidence of IRR was overlooked and that it should be over 20% in the case of optimal VCUG procedure (23). The incidence of IRRs, diagnosed by ceVUS, was not known for a long time. Some authors reported a case series of four children (2 girls and 2 boys) with IRR-associated VURs (24). No one of the two boys from that case series had a posterior urethral valve (PUV). Recently, three articles reported that the incidence of IRR was 12–49.5% of all VURs diagnosed by ceVUS (19, 25, 26).

Since ceVUS does not bear any radiation dose for the patient and shows high safety and sensitivity, recently it has become the method of choice for the diagnostics of VUR in many hospitals worldwide (27, 28). It is also known that IRR preferably appears in the parts of the kidney with compound papillae types II and III. Therefore, ceVUS became the method of choice for detecting that kind of papillae in patients with VUR, showing their natural distribution (15, 18, 19). IRRs are distributed more frequently in the upper and lower renal segments, and significantly less in the middle segment, just like compound papillae types II and III, which was reported in one study (19). Since IRR was assigned as a potential place of parenchymal damage in the previous studies (17, 22, 23, 29, 30), we expect more parenchymal changes at the places of IRR in this study. Moreover, since a higher number of IRRs can be detected by ceVUS than by VCUG (19, 26), we expect some other signs of parenchymal damage, not only scars, diagnosed by DMSA scan in renal regions affected by IRR in patients diagnosed by ceVUS.

The aim of this study was to present the number and positions of IRRs in kidneys affected by VURs in boys and girls, and the possible parenchymal changes in kidneys with IRR-associated VURs, diagnosed by ceVUS and DMSA scan. Since the IRR is several times more detectable by ceVUS than by VCUG, we expect more parenchymal defects due to IRR that can be attributed to IRR in patients with IRR-associated VUR.

In this study, we aim to describe even subtle parenchymal defects, not only scars in the kidneys with IRR, diagnosed by ceVUS and by using a DMSA scan.

Our aim is also to estimate the role of recurrent UTI, VUR, and IRR associated with VUR in the occurrence of parenchymal defects in the kidneys by using a DMSA scan.

## Patients and Methods

In this retrospective study that lasted from March 2017 to July 2021, we included 102 patients that were examined by ceVUS first and by ^99m^Tc-DMSA scintigraphy (DMSA scan) subsequently. Inclusion criteria for the study were recurrent febrile UTI or the first febrile UTI with abnormalities on renal ultrasound.

Patients without VUR, diagnosed by ceVUS, were not included in the study due to ethical reasons, but uretero-renal units (URU) without VUR from the contralateral side of diagnosed VUR in patients with diagnosed VUR have been analyzed by DMSA scan and included in the study as a control group.

Out of expected 204 URUs, 18 URUs were excluded from the study for the following reasons: duplex channel system (11 URUs), multicystic dysplasia (2 URUs), pelvicalyceal dilation and megaureter (2 URUs), non-functioning kidney (1 URU), renal ectopy (1 URU), and renal agenesis (1 URU). Altogether, we analyzed 186 URUs. Out of that number, 47 URUs did not have VURs, while 139 had VURs of all grades. The URUs with VURs were divided into three groups regarding the VUR grade: (1) non-dilating VURs (grades I–II), (2) mildly dilating VUR (grade III), and (3) dilating VURs (grades IV–V). Two boys from the study had PUV. Altogether, we analyzed three URUs because one boy was unilaterally nephrectomized. To perform a better analysis of changes in the renal parenchyma, we also divided kidneys into three separate parts, namely, the lower renal segment, middle renal segment, and upper renal segment.

The group of 47 URUs (141 renal segments) without VUR served as the control group (group A). The group of 139 URUs (417 renal segments) with VUR was additionally divided into two subgroups: 73 without IRR (219 renal segments) (group B) and 66 with IRR (198 renal segments) (group C). All patients from the studied groups underwent a DMSA scan. The URUs with IRRs were not additionally classified according to the size of the IRR.

### Contrast-Enhanced Voiding Urosonography Procedure

**FIGURE 1:**
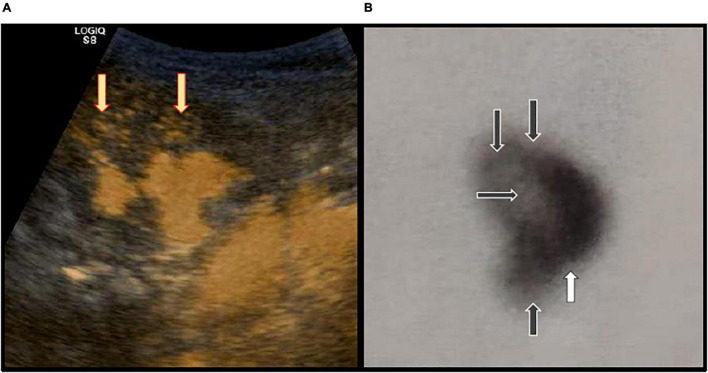
**(A)** Contrast-enhanced voiding urosonography (ceVUS) of the right kidney. The intrarenal reflux (IRR) is visible in the upper and middle renal segments (indicated by yellow arrows). **(B)** DMSA scan of the right kidney shows reduced DMSA signals (indicated by black arrows) in the upper, middle, and lower renal segments (indicated by black arrows), and a scar in the lower renal segment (indicated by white arrow).

Although the VCUG is still the gold standard for the detection of VUR in some hospitals, ceVUS is a standard procedure for the same indications in our hospital due to the absence of radiation, high sensitivity, and safety if performed according to current recommendations (27, 28, 31–34). The ultrasound contrast agent (UCA) that we used consisted of an active substance, sulfur hexafluoride in the form of microbubbles (SonoVue, Bracco, Italy). The procedure lasts 10–20 min and can be repeated several times, if necessary. The procedure starts with the catheterization of the patient’s bladder with a 6F–8F feeding tube or hydrophilic catheter. When the bladder is completely emptied, then the UCA solution is prepared by injecting 0.5 ml of UCA into 250 ml of the saline bag and instilled into the urinary bladder through the catheter under the pressure of 70–100 cm column of water. The bladder is filled until functional bladder capacity is reached or until the child starts to void. ceVUS was followed by Ultrasound machine GE Logiq S8 by probes C 2–9 and 9 L, using B-mode and harmonic imaging with a mechanical index of 0.06–0.08. During the procedure, two experienced pediatric nephrologists inspected the kidneys and bladder every 10–20 s and recorded digital images and video clips, if necessary. The results were concluded consensually. The appearance of IRR looks like a “smoke-like” extension of UCA, spreading from the renal calyces through the papillary ducts to the renal parenchyma. Besides the appearance of IRR, we also recorded the exact locations of IRR, distinguishing the occurrence of IRRs in the upper, middle, or lower renal segments, or in combined locations (Figure 1).

### DMSA Scan Procedure

Every patient from the study underwent a DMSA scan following guidelines from the European Association of Nuclear Medicine (35). The DMSA scan is a nuclear imaging procedure that identifies the uptake of DMSA in the kidney parenchyma. Usually, the DMSA scan is performed approximately 6 months after the first febrile UTI. The procedure is started with an i.v. injection of ^99m^Tc-DMSA to the patient in the supine position. Doses of radioisotope are measured according to the patient’s body surface. The images are taken from both sides (anterior and posterior positions of the patient). Images are made by a double-headed gamma camera with a low-energy, high-resolution collimator. Images of both kidneys are made with a total of 300,000–500,000 counts with a matrix of 128 × 128. A DMSA scan was normal if there was a homogenous uptake of the radiotracer. Three independent specialists in nuclear medicine estimated visual measurements in DMSA scans. They were not aware of the results of the previously performed ceVUS or renal ultrasonography. They were told to analyze upper, middle, or lower renal segments independently in terms of the intensity of DMSA uptake and the possible presence of parenchymal scars. Only the findings that were confirmed by at least two observers were taken into consideration (Figure 1). The parenchymal scar was defined as a decreased uptake and inhomogeneous distribution of DMSA together with a deformed renal outline over the affected part of the kidney.

### Statistical Analysis

We used the IBM SPSS Statistics program for our statistical data analysis. As the Shapiro–Wilk test indicated a statistically significant deviation from a normal distribution of numeric variables, the median and interquartile ranges were used. Analysis of the statistical significance of differences in a numeric variable between groups was performed using the Mann–Whitney test. Categorical variables were presented as numbers and percentages. The statistical significance of the differences in categorical variables was calculated using the Chi-square test. Logistic regression was also used. Statistical significance was set at *p* < 0.05. All confidence intervals were given at 95%.

## Results

### The Data Regarding the Sex

In this study, we examined 102 patients, 62 (61%) girls and 40 (39%), boys, with a median age of 14.25 months (IQR: 6–26 months; range: 1–91 months) at the VUR diagnosis. The median age at the time of VUR diagnosis in girls was 16.5 months (IQR: 8.9–35.5; range: 3–91) and 8.5 months (IQR: 4–19.5; range: 1–66.5) in boys. The median age at the time of diagnosis was significantly higher in girls than in boys (*Z* = 3.9; *p* = 0.001). In 70 analyzed URUs in boys, 36 (51.4%) had IRR, while out of 116 analyzed URUs in girls, 30 (25.9%) had IRR. The share of IRR was 2.0 times higher in boys than in girls (χ^2^ = 12.4; *p* < 0.001). Out of 70 URUs in boys, 31 (44.3%) had dilating VUR, while out of 116 URUs in girls, 28 (24.1%) had dilating VURs. The share of dilating VURs was 1.8 times higher in boys than in girls (χ^2^ = 7.7; *p* = 0.005).

### The Distribution of Vesicoureteral Reflux Grades Among the Groups of Uretero-Renal Units With Vesicoureteral Reflux

Out of 139 URUs with VURs, 80 (57.5%) had VURs of grades I and II, 43 (30.9%) had VURs of grade III, while 16 (11.5%) had VURs of grades IV and V (Table 1). Among them, 73 (52.5%) did not have IRR (group B), while 66 (47.5%) had IRR (group C). Group B consisted of 66 (90%) VURs from grades I and II and 7 (10%) VURs from grade III. Group C consisted of 14 (21%) VURs from grades I and II, 36 (55%) VURs from grade III, and 16 (24%) VURs from grades IV and V.

**TABLE 1:** The number and percentage of URUs with IRR regarding the location of IRR, and the grade of VUR.

		Grade of VUR			
IRR	Total *n* (%)	I and II *n* (%)	III *n* (%)	IV and V *n* (%)	*P* *
Lower segment					
IRR No	97 (70)	73 (91)	19 (44)	5 (31)	< 0.001
IRR Yes	42 (30)	7 (9)	24 (56)	11 (69)	
Middle segment					
IRR No	117 (94)	78 (97)	32 (74)	7 (44)	*P* < 0.001
IRR Yes	22 (16)	2 (3)	11 (26)	9 (56)	
Upper segment					
IRR No	85 (61)	69 (86)	14 (33)	2 (12)	*p* < 0.001
IRR Yes	54 (39)	11 (14)	29 (67)	14 (88)	

URU, Uretero-renal unit, IRR, Intrarenal reflux. *χ^2^ test.

### The Distribution of Intrarenal Reflux in the Group C Regarding the Location in the Kidney

Out of 66 URUs from group C, a total number of 118 IRRs were registered in different segments of the kidney. IRRs were distributed in 42 (63.6%) *lower renal segments*, 22 (33.3%) *middle segments*, and 54 (81.8%) *upper segments* alone, or in combinations, with significant differences between the groups (χ^2^ = 13.3, *p* = 0.001). In that group, IRRs appeared 2.5 times more frequent in the *upper renal segments* than in the *middle segment*, while the IRRs in the *lower segment* appeared 1.9 times more frequent than in the *middle segment* (Table 1). Although the incidence of IRR was higher in the *upper segment* than in the *lower renal segment*, there was no statistically significant difference between those two groups (χ^2^ = 1.5, *p* = 0.221) (Table 1).

### The Relationship Between Intrarenal Reflux and Vesicoureteral Reflux Grade

In this study, we found a statistically significant correlation of IRR incidence with the grade of VUR in the *lower renal segment* (χ^2^ = 42; *p* < 0.001), in the *middle segment* (χ^2^ = 33; *p* < 0.001), and in the *upper segment* (χ^2^ = 52; *p* < 0.001). The share of IRRs was 7.7 times higher in VURs of grades IV and V than in the group of VURs of grades I and II, while the share of IRR in VURs of grade III was 6.2 times higher than in the group of VURs of grades I and II in the *lower renal segment*, 19 times higher in VURs of grades IV and V than in the group of VURs of grades I and II, while the share of IRR in VURs of grade III was 8.7 times higher than in the group of VURs of grades I and II in the *middle renal segment*, and 6.3 times higher in VURs of grades IV and V than in the group of VURs of grades I and II, while the share of IRR in VURs of grade III was 4.3 times higher than in the group of VURs of grades I and II in the *upper renal segment*.

Between the groups of VURs of grades IV and V and the group of VURs of grades III, there was no statistically significant difference regarding the existence of IRR in the *lower segment* (*χ^2^ = 0.361, *p* = 0.548), in the *middle segment* (χ^2^ = 0.361, *p* = 0.057), and the *upper segment* (χ^2^ = 1.5, *p* = 0.226).

The probability for the occurrence of IRR increased 3.2 times (95% CI: 2–4.7; *p* < 0.001) with each increase in VUR grade in the *lower renal segment*, 3.7 times (95% CI: 2.1–6.5; *p* < 0.001) in the *middle renal segment*, and 3.6 times (95% CI: 2.4–5.3; *p* < 0.001) in the *upper renal segment* (Table 1).

### The Intensity of the DMSA Signal Among Groups A, B, and C Regarding the Renal Segments

The incidence of reduced DMSA signal was statistically different between groups A, B, and C in the *lower renal segment* (χ^2^ = 11.8, *p* = 0.003), in the *middle renal segment* (χ^2^ = 13.8, *p* = 0.001), and in the *upper renal segment* (χ^2^ = 11.2, *p* = 0.004) (Table 2). The incidence of reduced DMSA signal in the *lower renal segment* was 6 times higher in group C than in group A and 3 times higher than in group B, while it was 6.5 times higher in group C than in group A and 3.7 times higher than in group B in the *middle renal segment*, and 2.2 times higher in group C than in group A and 1.7 times higher than in group B in the *upper renal segment* (Table 2).

**TABLE 2:** The number and percentage of URUs with normal or reduced intensity of DMSA signal regarding the location in the kidney in the group’s A, B, and C.

The intensity of the DMSA signal	Total *n* (%)	Group A *n* (%)	Group B *n* (%)	Group C *n* (%)	*P* *
Lower segment					0.001
Normal signal	161 (87)	45 (96)	67 (92)	49 (74)	
Reduced signal	25 (13)	2 (4)	6 (8)	17 (26)	
Middle segment					0.001
Normal signal	167 (90)	46 (98)	69 (94)	52 (79)	
Reduced signal	19 (10)	1 (2)	4 (6)	14 (21)	
Upper segment					0.004
Normal signal	119 (64)	36 (77)	51 (70)	32 (48)	
Reduced signal	67 (36)	11 (23)	22 (30)	34 (52)	

Group A = URU without VUR; Group B = URU with VUR and without IRR; Group C = URU with VUR and IRR. *χ^2^ test.

Between groups A and B, there was no statistically significant difference regarding the incidence of reduced DMSA signal for the lower renal segment (χ^2^ = 0.399, *p* = 0.528), the middle segment (χ^2^ = 0.184, *p* = 0.668), and for the upper renal segment (χ^2^ = 0.356, *p* = 0.551) (Table 2).

Because of that, we fussed groups A and B and considered groups A and B as one unique group (A + B).

### The Intensity of the DMSA Signal Between the Group A + B and Group C Regarding the Renal Segments

The incidence of reduced DMSA signal was statistically different between group A + B and group C in the *lower renal segment* (χ^2^ = 11.7; *p* = 0.001), the *middle segment* (χ^2^ = 11.7; *p* = 0.001), and the *upper renal segment* (χ^2^=9.6; *p* = 0.002). The reduced uptake of the DMSA signal was 3.2 times higher in group C than in group A + B in the *lower segment*, 5 times higher in the *middle segment*, and 1.9 times higher in the *upper segment*.

The probability for the occurrence of reduced DMSA signal was 4.8 times higher in group C than in group A + B in the *lower segment* (odds ratio: 4.8; 95% CI: 2–12; *p* = 0.001), 6.2 times higher in the *middle segment* (odds ratio: 6.2; 95% CI: 2–18; *p* = 0.001), and 2.8 times higher in the *upper segment* (odds ratio: 2.8; 95% CI: 1.5–5.2; *p* = 0.002) (Table 3).

**TABLE 3:** The number and percentage of URUs with normal or reduced intensity of DMSA signal regarding the location in the kidney in the groups A + B, and C.

The intensity of the DMSA signal	Total *n* (%)	Group A + B *n* (%)	Group C *n* (%)	*P* *	Odds ratio (95% CI)	*P* ^†^
Lower segment				0.001	4.8 (2–12)	0.001
Normal signal	161 (87)	112 (93)	49 (74)			
Reduced signal	25 (13)	8 (7)	17 (26)			
Middle segment				0.001	6.2 (2–18)	0.001
Normal signal	167 (90)	115 (96)	52 (79)			
Reduced signal	19 (10)	5 (4)	14 (21)			
Upper segment				0.002	2.8 (1.5–5.2)	0.001
Normal signal	119 (64)	87 (72)	32 (48)			
Reduced signal	67 (36)	33 (28)	34 (52)			

*Group A + B = URUs with or without VUR and without IRR; Group C = group of URUs with VUR and with IRR.*

*χ^2^ test; P^†^ logistic regression.

### The Intensity of the DMSA Signal in Group C Regarding the Vesicoureteral Reflux Grades

There was no statistically significant relationship between the occurrence of reduced DMSA signal and the grade of VUR in the lower renal segment (χ^2^ = 1.4; *p* = 0.496), in the middle segment (χ^2^ = 2.6, *p* = 0.266), and in the upper renal segment (χ^2^ = 0.051; *p* = 0.975) in group C (Table 4).

**TABLE 4:** The intensity of the DMSA signal in the lower, middle, and upper renal segment regarding the VUR grade in the group C.

	Group C	VUR grade			
The intensity of the DMSA signal	Total *n* (%)	I and II *n* (%)	III *n* (%)	IV and V *n* (%)	*P* *
Lower segment					0.496
Normal signal	49 (74)	12 (86)	25 (69)	12 (75)	
Reduced signal	17 (26)	2 (14)	11 (31)	4 (25)	
Middle segment					0.266
Normal signal	52 (79)	13 (93)	26 (72)	13 (81)	
Reduced signal	14 (21)	1 (7)	10 (28)	3 (19)	
Upper segment					0.975
Normal signal	32 (48)	7 (50)	17 (47)	8 (50)	
Reduced signal	34 (52)	7 (50)	19 (53)	8 (50)	

*χ^2^ test.

### The Analyses of Decreased DMSA Signal Between Groups A, B, and C

Out of 47 URUs from group A (141 positions), the reduced signal was noticed in 11 URUs in 14 positions (9.9%). Out of 73 URUs from group B (219 positions), the reduced signal was noticed in 22 URUs in 32 positions (14%). Out of 66 URUs from group C (198 positions), the reduced signal was noticed in 36 URUs in 65 positions (32%).

Out of all 118 IRRs from group C, 45 (38.1%) had reduced intensity of signal, and 73 (61.9%) had normal signal.

Between groups A and B, there was no statistically significant difference regarding the numbers of renal parts affected by reduced signal (χ^2^ = 1.68; *p* = 0.194), while that difference was significant between group A + B and group C (χ^2^ = 32.2; *p* < 0.001).

In group A + B, the reduced signal was present in 46 positions (12%), while that number was present in 46 (32%) in group C, which was 2.7 times higher than in group A + B.

The probability of the occurrence of decreased signal in group C was 3.34 times higher than in group A + B (OR: 3.34; 95% CI: 2.2–5.1).

### Parenchymal Scars

Out of a total number of 11 parenchymal scars that were found in all three groups (A, B, and C), 2 belonged to group B, 9 belonged to group C, and 0 belonged to group A.

The mean elapsed time between the first febrile UTI and DMSA scan was 8 months (median: 8 months; min-max: 1–66 months).

## Discussion

This study showed the exact number of IRRs in patients with recurrent UTI and VUR and analyzed the possible effects of VUR and IRR on renal parenchyma using the ceVUS and DMSA scans.

### The Specific Characteristics of Analyzed Patients Regarding the Sex

Although this study altogether included 61% of girls, the share of boys with IRR was 55% of all examined patients. That percentage was consistent with some other studies (23, 25, 26), while in the study by Kim S. W et al. that percentage was even 98% (21).

Boys from our study had a significantly higher percentage of IRR-associated VURs (51.4%) and a higher percentage of dilating VURs (44.3%) compared to girls, with 25.9% and 24.1%, respectively. Moreover, the boys had earlier clinical presentations than the girls. All aforementioned factors could be the predisposing factors for the impairment of renal parenchyma, as was shown earlier (23). Therefore, we conclude that boys are at special risk for the development of parenchymal renal injury during exposure to VUR and IRR.

In this study, we had 2 boys with PUV. One of them was unilaterally nephrectomized. In that group, we analyzed three URUs and got three VURs. Out of all three URUs, one was of grade V with IRR, one of grade III without IRR, and one of grade II without IRR, which was not enough to make conclusions.

In this study, we were not focused on the diagnosis of PUV using ceVUS because our patients were highly suspected to have PUV because of characteristic clinical appearance and US examination before ceVUS. That suspicion was enough for the insertion of a urinary balloon catheter and subsequently undertaken diagnostic and therapeutic cystoscopy. The ceVUS examinations were done after the PUV was already diagnosed. To our mind, the diagnosis of PUV could be achieved easily if we combined the characteristic clinical appearance, the ultrasound of the urinary system, together with ceVUS, because the US is an integral part of the ceVUS procedure. Like some other authors, we do not think that the VCUG is an obligatory method for the diagnosis of PUV (19, 36).

Out of all the detected VURs, the percentage of detected IRRs in this study was 47.5%. If we exclude the results of Cvitkovic Roic et al. who reported 12% IRR, other studies also showed a higher incidence of IRR on ceVUS, which is a much higher incidence than in patients examined by VCUG, which has been reported to be 1–11% (17, 19, 20, 22, 25, 26).

These results are not surprising because some authors pointed out many possible difficulties regarding the protocol of VCUG. They claimed that the number of IRRs should be much higher and that IRRs sometimes represent an elusive phenomenon that demands very close follow-up during the procedure of VCUG (23). In the case of ceVUS, an observer can follow up on the entire diagnostic procedure in real-time and record all phases of the examination without exposure to X-rays.

The second interesting finding from this study is that 90% of VURs without IRR belonged to a non-dilating group of VURs, while 79% of IRR-associated VURs belonged to the group of dilating VURs. This has been noticed earlier in the studies that used VCUG and in some recent studies that used ceVUS (19, 21, 23, 25, 26). Moreover, this study, like the study by Simičić Majce A. et al. showed that the increased incidence of IRR is in accordance with the increase in VUR grade in all renal segments (19).

Thus, the occurrence of IRR is a characteristic of the group of dilating VURs. However, we also found 21% of non-dilating VURs that were associated with IRR in the group of IRR-associated VURs. Similar figures were noticed in the study of Kim SW et al. (14%), where VCUG was used for the detection of VUR. In contrast, Schneider KO et al. did not find any IRR in the group of non-dilating VURs. In our previous study, performed by ceVUS, we found 15% of IRRs in the group of non-dilating VURs, while Cvitković Roić et al. found only 3.5% (19, 25).

### The Role of DMSA Scan in the Study of IRRs

A previous study reported that the number of parenchymal changes differed significantly between the groups of kidneys without VUR from those with VURs regarding the intensity of DMSA signal and the scar formation in patients examined by VCUG and DMSA scan. Moreover, that study showed significantly more parenchymal changes in the group of dilating VURs than in the group of non-dilating VURs and in the group without VURs. They suggested that kidneys without or with low-grade VURs represent the low-risk group regarding the development of renal parenchymal damage, while the group of dilating VURs represents the high-risk group and should be followed more carefully regarding the development of kidney damage (11). The changes in DMSA uptake in this study can be considered permanent because the mean time elapsed between the first febrile UTI and the DMSA scan was 8 months, which is more than 3–6 months that was recommended by Jakobsson B et al. and Polito C et al. (37, 38).

In this study, we used a DMSA scan to detect the parenchymal injuries as possible late consequences of IRR. Our study showed that IRRs are distributed mostly in polar areas, while they are significantly less located in the middle segment, which corresponded to the natural distribution of composed papillae types II and III, as also noted in some previous reports (15, 18, 19).

This study showed a significantly higher incidence of reduced DMSA signal in the URUs with IRR than in the groups of URUs without VURs and with VURs but without IRR. Moreover, the groups without VURs and groups with VURs but without IRR did not differ regarding the incidence of reduced DMSA signal. That fact indicates that the probability of starting parenchymal injury is much higher in the URUs with IRR than in the other two groups. That was also proved in the study by Kim S. W et al. where the sites of IRRs detected by VCUG had photon defects in 76.3% of cases. In addition, they reported that sites of IRR tended to develop scars in 65.2% over time, while the process of scar formation could be slowed down by using surgical therapy (21). However, Kim SW et al. analyzed only a small number of IRRs, i.e., the small percentage of patients with VUR, because the VCUG can show a lower number of IRRs, probably those connected with the highest grades of VUR. In our study using the ceVUS and DMSA scans, a large number of IRRs were presented with a wider spectrum of parenchymal changes and not only by scars. In the group of patients with IRR from this study, we found no difference in changes in DMSA signal between the grades of VUR. That means that once IRR exists, the changes in renal parenchyma will be present regardless of the grade of VUR.

The analysis of the number and locations of reduced DMSA signals in the affected kidneys in the different groups of patients showed that the group with IRR-associated VURs was significantly more affected by reduced DMSA signals than the group without VUR and the group with VUR but without IRR. That is the direct and undoubtful proof that IRR plays a significant role in the process of renal parenchymal injury. IRR could facilitate the transmission of infective agents and allow the water hammer effect of backflow of urine through the compound papillae types II and III, causing consequences such as scars, pressure-related damage of papillae, delay of kidney growth, and finally shrunken kidneys (16, 39).

Our study has also shown that only 38% of positions affected by IRRs had decreased DMSA signal, which gives us the hope that there are still 62% of remaining unaffected positions, which allows us to act by preventive and therapeutic measures in order to prevent renal injury.

Although we found the expected small number of scars in the renal parenchyma, we did not find any scars in the URUs without VUR and only two scars in the group with VUR without IRR. The majority of the nine scars have been found in the group of URUs with IRR. This finding is in accordance with data from the previous studies done by VCUG, showing that only the biggest IRRs can be detected by VCUG, and those are usually connected with scar formation. Since ceVUS detects more IRRs, we can find the subtle, starting changes in the renal parenchyma, not only scars, allowing us to start with treatment and that way to prevent renal damage. All that indicates that ceVUS has become a sensitive and useful diagnostic method, which could point to optimal moments for an early therapeutic and preventive approach to patients with VUR and IRR. Therefore, in our opinion, we suggest that IRR should be incorporated in the future classification of VURs because of clear evidence that kidneys with IRR-associated VURs belong to a risk group of VURs and should be treated more actively than those without IRR, as was previously mentioned in some other reports (19, 23).

## Conclusion

The parenchymal changes are much more developed in URUs with IRR-associated VURs and in the renal parts associated with IRR than in those without IRR.

Even though the parenchymal damage is more expected in the higher grades of VUR due to a higher incidence of IRR, that damage is also expected in the VURs of lower grades accompanied by IRR.

VURs of higher grades and those with IRR have an early clinical presentation.

Boys with VUR have an earlier clinical presentation than girls due to the fact that they have significantly higher grades of VUR with a higher proportion of IRRs.

According to these results, we can divide VURs into two additional groups: those with IRR, with a higher expectance of parenchymal damage, and those without IRR, with a less chance of parenchymal damage.

## Data Availability Statement

The original contributions presented in this study are included in this article/supplementary material, further inquiries can be directed to the corresponding author.

## Ethics Statement

The studies involving human participants were reviewed and approved by the Ethical Board of University Hospital in Split. Written informed consent to participate in this study was provided by the participants’ legal guardian/next of kin.

## Author Contributions

ASM, AA, VČ, DB, MB, IZ, AB, AP, MS-B, KV, and MS: conceptualization, methodology, validation, formal analysis, investigation, data curation, writing—original draft, and approved for submitted version.

## Conflict of Interest

The authors declare that the research was conducted in the absence of any commercial or financial relationships that could be construed as a potential conflict of interest.

## Publisher’s Note

All claims expressed in this article are solely those of the authors and do not necessarily represent those of their affiliated organizations, or those of the publisher, the editors and the reviewers. Any product that may be evaluated in this article, or claim that may be made by its manufacturer, is not guaranteed or endorsed by the publisher.

## References

[1] Bailey RR. Vesicoureteric reflux in healthy infants and children. 1st ed. In: Hodson CJ Kincaid Smith P editors. *Reflux Nephropathy.* New York, NY: Masson (1979). p. 59–61.

[2] Lebowitz RL Olbing H Parkkulainen KV Smellie JM Tamminen-Mobius TE. International systemof radiographic grading of vesicuureteric reflux. International reflux study in children. *Pediatr Radiol.* (1985) 15:105–9. 10.1007/BF02388714 3975102

[3] Darge K Troeger J. Vesicoureteral reflux grading in contrast enhanced voiding urosonography. *Eur J Radiol.* (2002) 43:122–8. 12127209 10.1016/s0720-048x(02)00114-6

[4] Saraga M Staniciæ A Markoviæ V. The role of direct radionuclide cystography in evaluation of vesicoureteral reflux. *Scand J Urol Nephrol.* (1996) 30:367–71. 10.3109/00365599609181312 8936625

[5] Snodgrass WT Shah A Yang M Kwon J Villaneuva C Taylor J Prevalence and risk factors for renal scars in children with febrile UTI and/or VUR: a cross-sectional observational study of 565 consecutive patients. *J Pediatr Urol.* (2013) 9:856–63. 23465483 10.1016/j.jpurol.2012.11.019 PMC3770743

[6] Mattoo TK Chesney RW Greenfield SP Hoberman A Keren R Mathews R Renal scarring in the randomized intervention for children with vesicoureteral reflux (RIVUR) trial. *Clin J Am Soc Nephrol.* (2016) 7:54–61. 10.2215/CJN.05210515 26555605 PMC4702233

[7] Brandstrom P Neveus T Sixt R Stokland E Jodal U Hansson S. The Swedish reflux trial in children: IV. Renal damage. *J Urol.* (2010) 184:292–7. 20494369 10.1016/j.juro.2010.01.060

[8] Guarino S Capalbo D Martin N Campana G Rambaldi PF Miraglia Del Giudice E In children with urinary tract infection reduced kidney length and vesicoureteric reflux predict abnormal DMSA scan. *Pediatr Res.* (2019) 87:779–84. 10.1038/s41390-019-0676-1 31726462

[9] Buonomo C Treves ST Jones B Summerville D Bauer S Retik A. Silent renal damage in dymptom-free siblings of children with vesicoureteral reflux: assessment with technetium Tc 99m dimercaptosuccinic acid scintigraphy. *J Pediatr.* (1993) 122:721–3. 10.1016/s0022-3476(06)80012-0 8388446

[10] Kosmeri C Kalaitzidis R Simou E. An update on renal acarring after urinary tract infection in children: whar are the risk factors? *J Pediatr Urol.* (2019) 15:598–603. 31591046 10.1016/j.jpurol.2019.09.010

[11] Arapović A Punda A Brdar D Čapkun V Bajo D Veljačić D Types of parenchymal changes diagnosed on DMSA scans of kidneys affected by different grades of vesicoureteral reflux. *Med Sci Monit.* (2021) 27:e929617. 10.12659/MSM.929617 33647007 PMC7934341

[12] Brodeur AE Goyer RA Melick W. A potential hazard of barium cystography. *Radiology.* (1965) 85:1080–4. 10.1148/85.6.1080 5892138

[13] Darge K Trusen A Gordjani N Riedmiller H. Intrarenal reflux: diagnosis with contrast-enhanced harmonic US. *Pediatr Radiol.* (2003) 33:729–31. 10.1007/s00247-003-1050-2 12928758

[14] Papadopoulou F Anthopoulou A Siomou E Efremidis S Tsamboulas C Darge K Harmonic voiding urosonography with a second-generation contrast agent for the diagnosis of vesicoureteral reflux. *Pediatr Radiol.* (2009) 39:239–44. 10.1007/s00247-008-1080-x 19096835

[15] Ransley PG Risdon RA. Renal papillary morphology and intrarenal reflux in the young pig. *Urol Res.* (1975) 3:105–9. 10.1007/BF00256030 1189137

[16] Ransley PG Risdon RA. The pathogenesis of reflux nephropathy. *Contrib Nephrol.* (1979) 16:90–7. 467074 10.1159/000402880

[17] Rolleston GL Maling TM Hodson CJ. Intrarenal reflux and the scarred kidney. *Arch Dis Child.* (1974) 49:531–9. 10.1136/adc.49.7.531 4852544 PMC1648904

[18] Hannerz L Wikstad I Johansson L Broberger O Aperia A. Distribution of renal scars and intrarenal reflux in children with a past history of urinary tract infection. *Acta Radiol.* (1987) 28:443–6. 2958060

[19] Simièić Majce A Arapović A Saraga-Babić M Vukojević K Benzon B Punda A Intrarenal reflux in the light of contrast-enhanced voiding urosonography. *Front Pediatr.* (2021) 9:642077. 10.3389/fped.2021.642077 33738272 PMC7960767

[20] Uldall P Frøokjaer O Kaas Ibsen K. Intrarenal reflux. *Acta Paediatr Scand.* (1976) 65:711–5. 10.1111/j.1651-2227.1976.tb18008.x 998230

[21] Kim SW Im YJ Hong CH Han SW. The clinical significance of intrarenal reflux in voiding cystourethrography (VCUG). *Korean J Urol.* (2010) 51:60–3. 10.4111/kju.2010.51.1.60 20414413 PMC2855467

[22] Cremin BJ. Observations on vesico-ureteric reflux and intrarenal reflux: a review and survey of material. *Clin Radiol.* (1979) 30:607–21. 10.1016/s0009-9260(79)80003-3 509863

[23] Schneider KO Lindemeyer K Kammer B. Intrarenal reflux, an overlooked entity – retrospective analysis of 1,166 voiding cysturethrographies in children. *Pediatr Radiol.* (2019) 49:617–25. 10.1007/s00247-018-04330-z 30683961

[24] Colleran GC Barnewolt CE Chow JS Paltiel HJ. Intrarenal reflux: diagnosis at contrastenhanced voiding urosonography. *J Ultrasound Med.* (2016) 35:1811–9. 10.7863/ultra.15.09056 27371375

[25] Cvitkovic-Roic A Turudic D Milosevic D Palcic I Roic G. Contrast-enhanced voiding urosonography in the diagnosis of intrarenal reflux. *J Ultrasound.* (2021) 25:89–95. 10.1007/s40477-021-00568-w 33635511 PMC8964875

[26] Kim D Choi YH Choi G Lee S Lee S Cho YJ Contrast-enchanced voiding urosonography for the diagnosis of vesicoureteral reflux and intrarenal reflux: a comparison of diagnostic performance with fluoroscopic voiding cystourethrography. *Ultrasonography.* (2021) 40:530–7. 10.14366/usg.20157 33887876 PMC8446490

[27] Riccabona M Avni FE Blickman JG Dacher JN Darge K Lobo ML Imaging recommendationsin pediatric uroradiology: minutes of the ESPR workgroup session on urinary tract infection, fetal hydronephrosis, urinary tract ultrasonography and voiding cystourethrography, Barcelona, Spain, june2007. *Pediatr Radiol.* (2008) 38:138–45. 10.1007/s00247-007-0695-7 18071685

[28] Ricabona M Avni FE Damasio MB Ording-Muller LS Blinckman JG Darge K ESPR uroradiology task force and ESUR pediatric working group-imaging recommendations in pediatric uroradiology, part V: childhood cystic kidney disease, childhood renal transplantation and contrast-enhanced ultrasonography in children. *Pediatr Radiol.* (2012) 42:1275–83. 10.1007/s00247-012-2436-9 23001574

[29] Rose JS Glassberg KI Waterhouse K. Intrarenal reflux and its relationship to renal scarring. *J Urol.* (1975) 113:400–3. 10.1016/s0022-5347(17)59492-6 1117509

[30] Fujimatsu A. Diagnosis of intrarenal reflux and its role in pathogenesis of reflux nephropathy in children. *Kurume Med J.* (2000) 47:109–14. 10.2739/kurumemedj.47.109 10948648

[31] Darge K. Voiding urosonography with ultrasound contrast agents for the diagnosis of vesicoureteric reflux in children. I. Procedure. *Pediatr Radiol.* (2008) 38:40–53. 10.1007/s00247-007-0529-7 17618429 PMC2292498

[32] Duran C Beltrán VP González A Gomez C del Riego J. Contrast-enhanced voiding urosonography for vesicoureteral reflux diagnosis in children. *Radiographics.* (2017) 37:1854–69. 10.1148/rg.2017170024 29019761

[33] Mane N Sharma A Patil A Gadekar C Andankar M Pathak H. Comparison of contrast-enhanced voiding urosonography with voiding cystourethrography in pediatric vesicoureteral reflux. *Turk J Urol.* (2018) 44:261–7. 10.5152/tud.2018.76702 29733800 PMC5937646

[34] Ntoulia A Back SJ Shellikeri S Poznick L Morgan T Kerwood J. Contrast-enhanced voiding urosonography (ceVUS) with the intravesical administration of the ultrasound contrast agent Optison™ for vesicoureteral reflux detection in children: a prospective clinical trial. *Pediatr Radiol.* (2018) 48:216–26. 10.1007/s00247-017-4026-3 29181582

[35] Piepsz A Colarinha P Gordon I Hahn K Olivier P Roca I Guidelines on 99mTc-DMSA scintigraphy in children. *Eur J Nucl Med.* (2001) 28:37–41. 11315615

[36] Giordano M Marzolla R Puteo F Scianaro L Carnigella DA Depalo T. Voiding urosonography as a first step in the diagnosis of vesicoureteral reflux in children: a clinical experience. *Pediatr Radiol.* (2007) 37:674–7. 10.1007/s00247-007-0499-9 17520246

[37] Jakobsson B Nolstedt L Svensson L Söderlundh S Berg U. 99m technetium dimercaptosuccinic acid scan in the diagnosis of acute pyelonephritis in children: relation to clinical and radiological findings. *Pediatr Nephrol.* (1992) 6:328–34. 10.1007/BF00869725 1343562

[38] Polito C Rambaldi PF Signoriello G Mansi L La Manna A. Permanent renal parenchymal defects after febrile UTI are closely associated with wesicoureteric reflux. *Pediatr Nephrol.* (2006) 21:521–6. 10.1007/s00467-006-0036-3 16491412

[39] Hodson CJ. Neuhauser lecture. Reflux nephropathy: a personal historical review. *AJR.* (1981) 137:451–62. 10.2214/ajr.137.3.451 7025597

